# Surgical delay in appendicitis among children: the role of social vulnerability

**DOI:** 10.3389/fped.2025.1591200

**Published:** 2025-07-02

**Authors:** Villa-Aguilar Estefany, Marín-Morales Karen, Ayala-Galvan Citlali, Gonzalez-Ortiz Ailema, Gonzalez-Zamora Jose Francisco

**Affiliations:** Centro de Investigación Traslacional, Instituto Nacional de Pediatría, Mexico City, Mexico

**Keywords:** complicated appendicitis, prehospital delay, access to surgical care, social vulnerability, health inequities, Hispanic pediatric, Mexican population

## Abstract

**Background:**

Appendicitis is the most common abdominal emergency in pediatrics and is suggested as a quality indicator for timely access to care in time-sensitive conditions. Despite advances in diagnostics, the incidence of complicated appendicitis (CA) remains high due to delays in pediatric surgical care, which can increase disease severity. Social vulnerability has been associated with surgical access, particularly in low- and middle-income populations. This study examines the relationship between surgical delay and social determinants of complicated appendicitis in children without social security in the Metropolitan Zone of México.

**Methods:**

This retrospective cohort study was conducted in a pediatric public hospital. Consecutive cases from 2018 to 2021 with confirmed appendicitis diagnoses were included. The primary outcome variable was the type of appendicitis (acute/complicated), while two independent variables were the time of evolution (patient and hospital timing) and socioeconomic factors associated with social vulnerability. A logistic regression analysis assessed the relationship between appendicitis type and covariates.

**Results:**

A total of 943 pediatric cases of appendicitis were included. Out of these, 62.67% presented CA, with a mean age 10 ± 4 years. 60.9% were well-nourished, and 16% had at least one comorbidity. Most subjects (76%) met at least two criteria for social vulnerability, and 74.7% of families held unskilled jobs. The median symptom-to-admission time was 2 days, with a median hospital-to-surgery time of 19.5 h (p25–p75). Prehospital delays affected 57.8% of cases (*n* = 545), and 39% (*n* = 365) experienced intrahospital delays. In the multivariable logistic regression, prehospital delay ≥48 h (OR 3.27, 95% CI 2.43–4.39) and children under 5 years (OR 1.76, 95% CI 1.09–2.84) were associated with higher odds of CA.

**Conclusion:**

The high frequency of CA in uninsured children at a public hospital is due to surgical delays of two or more days. Social vulnerabilities, as observed globally, hinder access to quality care. Thus, appendicitis should be recognized as both a medical and social issue, requiring a comprehensive approach that addresses social vulnerability.

## 1 Introduction

Appendicitis is the most common abdominal emergency in pediatrics (1, 2), where age and delays in accessing healthcare are the most consistent clinical risk factors influencing the disease's natural progression (3). Consequently, some authors have proposed it as a key quality indicator for access to emergency care for time-dependent conditions (4, 5).

Despite technological advances and substantial progress in the diagnostic evaluation of children with suspected appendicitis, the incidence of complicated appendicitis (CA) has not declined over time (6, 7). Access to pediatric surgical care is often associated with delays in diagnosis and treatment, which can lead to increased disease severity (8, 9). Among these factors, surgical delay is identified as the primary risk factor for complicated cases (10).

The disparity in access to surgical care is linked to outcomes in pediatric appendicitis. Barriers related to this disparity in healthcare systems are evaluated through social vulnerability, a set of standardized indexes based on social and individual risk indicators tailored to each population (11). Over the years, these indexes have been strongly associated with access to surgical care (10). According to the International Labor Organization, there is a global crisis due to the lack of social protection (12), which disproportionately impacts low- and middle-income countries (13). This situation creates barriers to accessing healthcare, such as out-of-pocket expenses, longer distances to medical facilities, limitations in the range of available services, low quality, poor acceptability of services, and long waiting times (14).

In Mexico, access to social protection is intricately linked to employment status, determining the public or private healthcare options available to individuals. Unfortunately, public healthcare services often fail to meet the needs of many users (15). Previous reports estimated that 40%–60% of the Mexican population lacks access to these services (15, 16), which are provided through the public healthcare system for the uninsured. Despite government efforts to reform this fragmented system, there has been no substantial documentation of changes in access to health services (17). In this context, it is important to investigate the frequency of complications in this surgical abdominal emergency among children of uninsured households. To address this, we analyzed the relationship between surgical delays and the social vulnerability of families, focusing on children diagnosed with CA who visited the emergency department of a hospital with a high volume of pediatric surgeries between 2018 and 2021.

## 2 Methods

### 2.1 Study design

This retrospective cohort study was conducted in a high-volume pediatric public hospital in Mexico City that primarily serves a population without social security. The primary sources of information were institutional databases and electronic records. The health research committees approved the protocol, registration number 069/2022. The report followed the Strengthening the Reporting of Observational Studies in Epidemiology (STROBE) guidelines (18).

### 2.2 Data source

Five institutional databases were utilized: the “Hospital Administration System” under the “Surgery Control” section, “Electronic Medical Record”, “Emergency Admission Register”, “Death Control”, and “Socioeconomic Study” from January 1, 2018, to December 31, 2021. The databases were linked, and the records were anonymized for data curation and analysis.

### 2.3 Study population

This study included subjects aged 1–18 years who met the following criteria: postoperative diagnosis of appendectomy and histopathological reports that included samples of the cecal appendix. Cases with a clinical diagnosis of appendicitis were confirmed through histopathological examination. Exclusion criteria included subjects with a diagnosis of a normal cecal appendix, interval appendectomy, or incomplete information. All patients diagnosed with appendicitis were taken to the operating room directly from the emergency department, and according to hospital protocols, all patients received at least one dose of antimicrobial treatment before surgery.

### 2.4 Outcomes

The primary outcome was the classification of appendicitis as either acute appendicitis (AA) or complicated appendicitis (CA). This classification was determined based on the intraoperative clinical diagnosis recorded in the postoperative notes of each record, following the criteria defined by Cameron (19) and validated by McKie (20). The histological report was used to confirm the diagnosis of a normal appendix.

Independent variables included the time of evolution and socioeconomic factors associated with social vulnerability. The evaluation of the time of evolution included patient time, defined as the days from initial presentation to admission to the emergency department as reported by the primary caregiver, and hospital time, defined as the hours from admission to the emergency department until admission to the operating room (1). Prehospital delay was considered when subjects had two or more days of symptoms (1, 8), while intrahospital delay was defined as taking 24 or more hours to arrive at the operating room (1, 21).

Socioeconomic factors associated with social vulnerability were assessed and evaluated, including: (1) location of residence; (2) employment status, categorized as unemployed, unskilled, and skilled workers (22). Two indicators of social deprivation proposed by “Consejo Nacional de Evaluación la Política de Desarrollo Social” CONEVAL (16): Inadequate housing quality and overcrowding, which occur if the home exhibits at least one of the following characteristics: (a) floors made of mud; (b) roofs constructed from cardboard sheeting or waste materials; (c) walls made of mud, reed, bamboo, palm, cardboard sheeting, metal, or other waste materials; (d) overcrowding, defined as more than two persons per room (16); and lack of basic housing services, defined as having at least one of the following characteristics: (a) water obtained from a well, river, lake, stream, or pipe; or piped water acquired by carrying it from another home or from a public tap or hydrant; (b) lack of sewage service or drainage connected to a pipe leading to a river, lake, sea, ravine, or crevice; (c) lack of electricity; (d) use of firewood or charcoal for cooking or heating food (16).

### 2.5 Study covariates

Other data included: admission date (year/month), sex (male/female), age group (young children under 5 years, school-age 6–11 years, and adolescents 12–18 years), shift of admission to the emergency department (morning/afternoon/night), nutritional status (underweight/normal/overweight/obesity according to BMI percentiles in growth charts proposed by the Centers for Disease Control and Prevention) (23), comorbidity (presence of any coexisting disease before the diagnosis of appendicitis, as reported in your medical record), and antimicrobial treatment prior to admission to the emergency department (yes/no, type, dose, and duration).

### 2.6 Statistical analysis

For the descriptive analysis of time variables, medians and ranges were calculated. Clinical and socioeconomic variables were described using frequencies and proportions. The comparison between clinical and socioeconomic variables and the type of appendicitis was made using the Chi-square test. We compared clinical characteristics, socioeconomic factors, and time delays between types of appendicitis using univariable logistic regression with CA as the dependent variable. Finally, a binary logistic regression analysis was performed to assess the association between the time of evolution, socioeconomic factors, and clinical characteristics and the likelihood of classifying appendicitis. Variables included in the model were selected based on biological plausibility, potential confounders, and a *p*-value of less than 0.20 in bivariate analysis. Odds ratios (ORs) with 95% confidence intervals (CIs) were calculated.

## 3 Results

### 3.1 Study cohort and case selection

**Figure 1:**
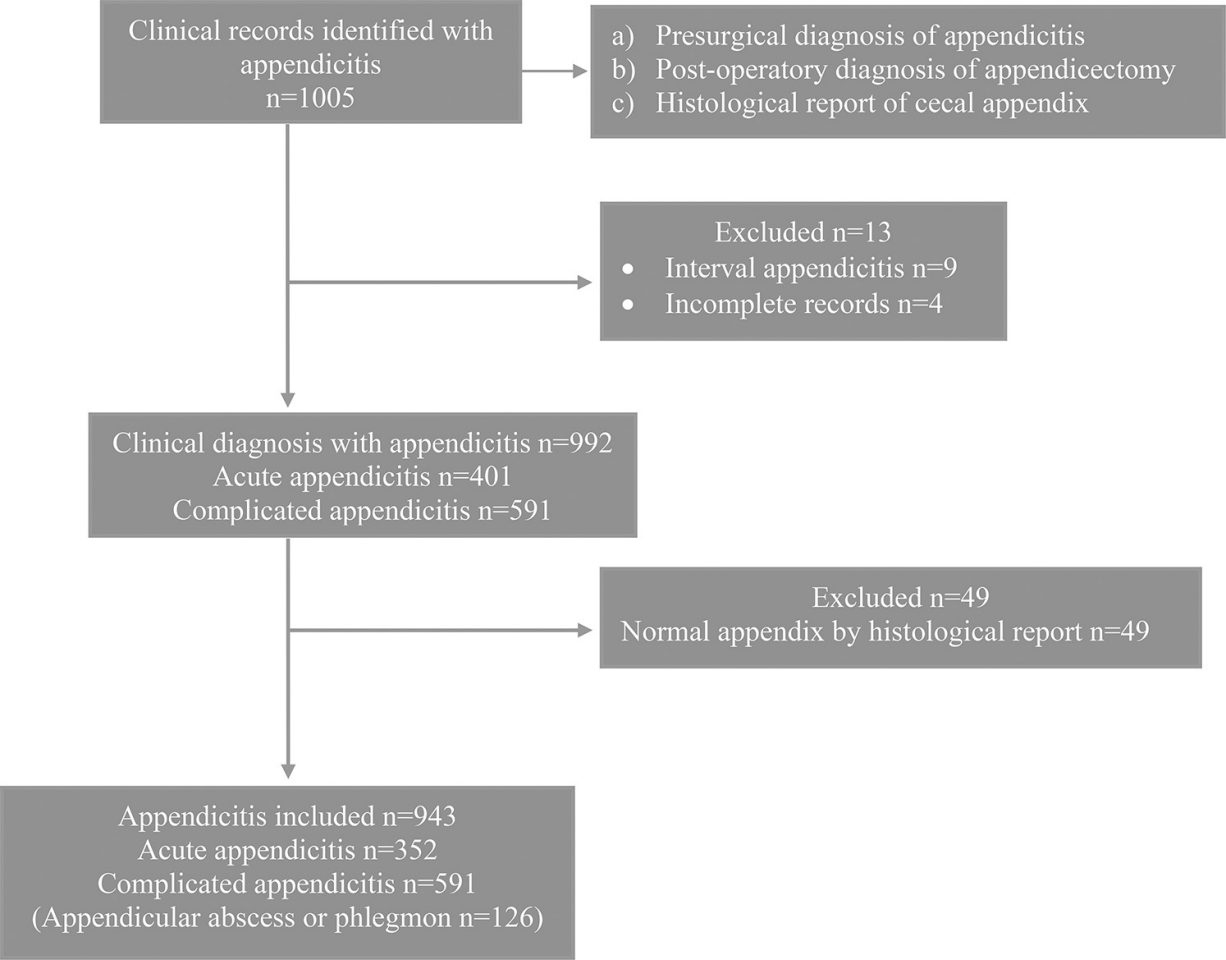
Flow diagram of patient selection in this observational study.

A total of 1,005 cases of appendicitis were identified between January 2018 and December 2021. An average of 21 appendectomies per month (±7.4) were performed, with a minimum of 16 (±8.3) in February 2020 and a maximum of 25.6 (±7.8) in June 2019. A total of 992 records of patients with appendicitis were included in the study, of which 401 were identified as having a diagnosis of AA. However, 49 were histologically identified as a normal appendix. Ultimately, 943 subjects were included in this study (Figure 1).

### 3.2 Population clinical characteristics

Of the 943 subjects included, 59.3% (560 subjects) were female, with a mean age of 10 years (±4). At the time of admission, 575 subjects (60.9%) exhibited a normal nutritional status. Comorbidities were identified in 150 subjects (16%), with the most common being global developmental delay 36 cases (3.8%), complete congenital heart defects 14 cases (1.5%), acute lymphoblastic leukemia in treatment 13 cases (1.4%), moderate persistent asthma 13 cases (1.4%), metabolic syndrome and obesity 8 cases (0.8%), systemic lupus erythematosus 5 cases (0.5%), Down syndrome 5 cases (0.5), autism spectrum disorder 4 cases (0.4%), central nervous system tumors 3 cases (0.3%), autoimmune diseases congenital 2 cases (1.3%) and various other morbidities (47 cases). Being in preschool or school was associated with a higher probability of CA, while the probability of having comorbidities was lower in patients with CA (OR 0.65, 95% CI 0.45–0.92) (Table 1).

**Table 1:** Clinical characteristics of patients with appendicitis.

Clinical characteristics	Total *n* = 943	Acute *n* = 352	Complicated *n* = 591	Unadjusted OR (95% CI)	*p*-value
Sex					
Female	560 (59)	212 (60)	348 (59)	1.0 (Ref)	0.684
Male	383 (41)	140 (40)	243 (41)	1.05 (0.80–1.38)	
Age group					
Young children	134 (14)	38 (11)	96 (16)	1.82 (1.18–2.81)	0.030
School-age	453 (48)	167 (47)	286 (49)	1.19 (0.90–1.59)	
Adolescent	356 (38)	147 (42)	209 (35)	1.0 (Ref)	
Nutritional condition					
Normal	575 (61)	211 (60)	364 (62)	1.0 (Ref)	0.356
Underweight	90 (10)	41 (12)	49 (8)	0.69 (0.44–1.08)	
Overweight	141 (15)	53 (15)	88 (15)	0.96 (0.65–1.4)	
Obese	137 (14)	47 (13)	90 (15)	1.11 (0.75–1.64)	
Comorbidity					
Yes	150 (16)	69 (20)	81 (14)	0.65 (0.45–0.92)	0.017
Year of procedure					
2018	249 (26)	96 (27)	153 (26)	1.0 (Ref)	0.464
2019	283 (30)	99 (28)	184 (31)	1.16 (0.81–1.66)	
2020	188 (20)	78 (22)	110 (19)	0.88 (0.60–1.30)	
2021	223 (24)	79 (23)	144 (24)	1.14 (0.78–1.66)	
Emergency department shift					
Morning	229 (24)	89 (25)	140 (24)	1.0 (Ref)	0.335
Afternoon	302 (32)	120 (34)	195 (31)	0.96 (0.67–1.37)	
Evening	412 (44)	143 (41)	282 (45)	1.19 (0–85–1.67)	

Values reported as *n* (%). Age group: Young children (under 5 years), school-age (6–11 years), and adolescents (12–18 years). Nutritional condition: CDC's BMI-for-age growth chart. Main comorbidities: 36 global development delay, 22 cancer, 14 congenital heart disease.

### 3.3 Sociodemographic characteristics and social vulnerability

We identified 919 families (97.5%) living in the Metropolitan Zone of Mexico City, a geographic area comprising Mexico City and neighboring municipalities in the states of Mexico and Hidalgo (an area of 7,180 km^2^ inhabited by 20 million people). 453 families (48%) resided in the five most densely populated municipalities with the highest poverty levels in Mexico City. Due to the primary caregiver's status of employment, the households were uninsured. At the time of hospital admission, we identified that in 189 families (21.6%), the primary caregiver was unemployed, and in 653 families (74.7%), caregivers were engaged in unskilled jobs (Table 2). Finally, 719 households (76%) met at least one criterion of social vulnerability (16).

**Table 2:** Socioeconomic factors associated with social vulnerability.

Socioeconomic factors	Total *n* = 943	Acute *n* = 352	Complicated *n* = 591	Unadjusted OR (95% CI)	*p*-value
Location of residence					
Mexico City	713 (76)	261 (74)	452 (77)	1.0 (Ref)	0.577
State of Mexico	206 (22)	80 (23)	126 (21)	0.90 (0.66–1.25)	
Others	24 (2)	11 (3)	13 (2)	0.68 (0.30–1.54)	
Employment status					
Unemployed	189 (21)	72 (24)	117 (21)	1.43 (0.67–3.04)	0.375
Unskilled labor	653 (75)	232 (73)	421 (76)	1.60 (0.78–3.26)	
Skilled labor	32 (4)	15 (3)	17 (3)	1.0 (Ref)	
Inadequate housing quality and overcrowding					
Yes	629 (72)	220 (69)	409 (73)	1.24 (0.91–1.68)	0.158
No	247 (28)	99 (31)	148 (27)	1.0 (Ref)	
Lack of basic services					
Yes	143 (16)	41 (13)	102 (18)	1.51 (1.02–2.23)	0.037
No	735 (84)	278 (87)	457 (82)		

Odds ratios (ORs) and 95% confidence intervals (CIs) were calculated using univariable logistic regression with complicated appendicitis as the outcome. * In 7% of the subjects, there wasn't complete information on Employment status *n* = 69, Housing quality and overcrowding=*n* = 67, and Access to basic services *n* = 61.

### 3.4 Role of delay among complicated appendicitis

**Figure 2:**
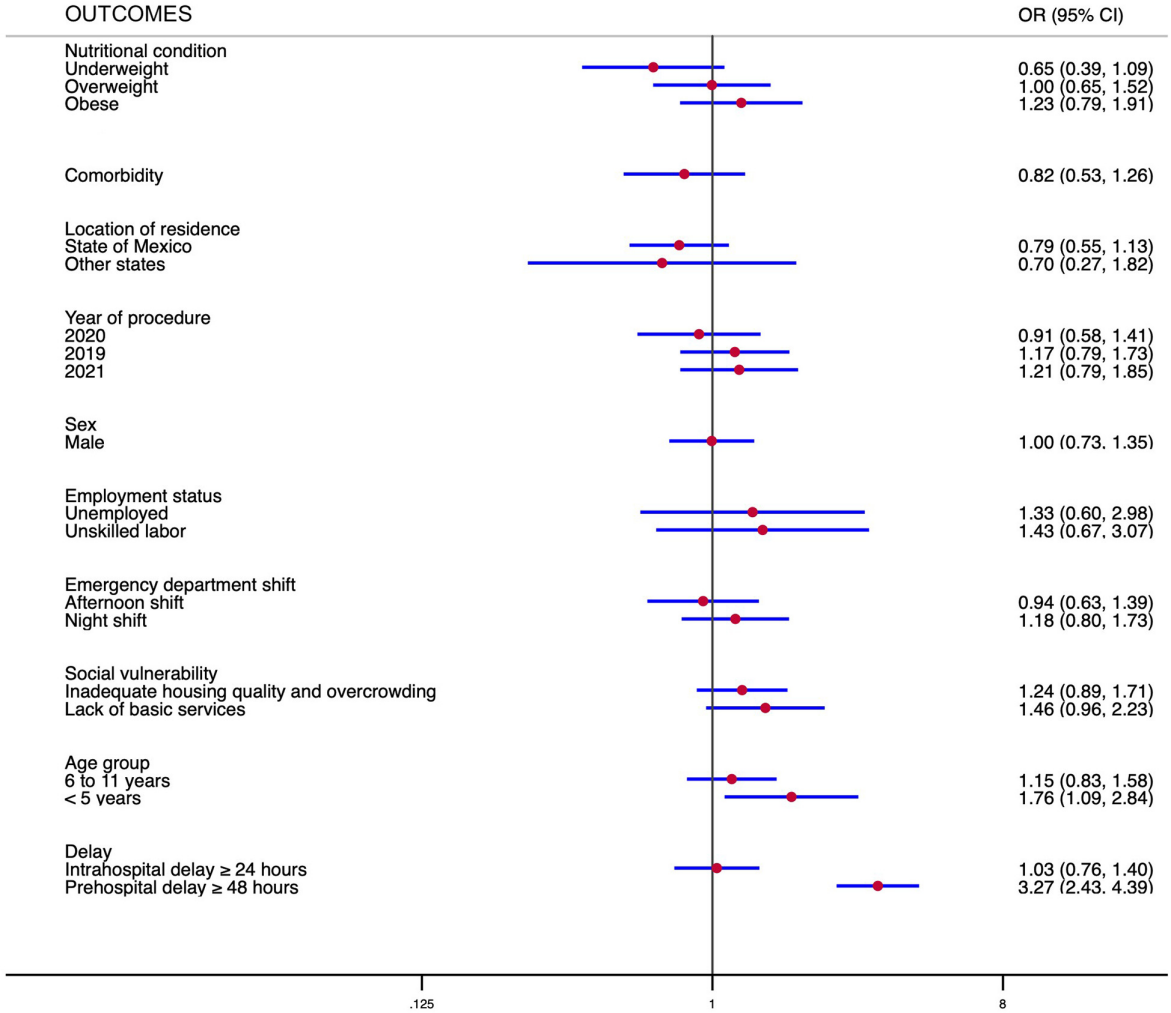
Multiple regression model examining the association between independent variables and covariates with complicated appendicitis. Foot note: Logistic regression, Log likelihood = −528.826, ch2(20) = 89.44, *p* < 0.000. Revealed significant findings: an increased risk for children under five years of age [odds ratio (OR) 1.76, 95% IC 1.09–2.84] and a prehospital delay of 48 h or more (OR 3.27, 95% CI 2.43–4.39).

Regarding the time of evolution of the population, the overall median time from the onset of symptoms to hospital admission (patient time) was 2 days (min 0–max 30). For patients with AA, the median was 1 day (min 0–max 8), while for CA, it was 2 days (min 0–max 30). The median time from hospital admission to the operating room (hospital time) was 19.5 h (min 1.1–max 137.2). For subjects with AA, it was 20.7 h (min 2.1–max 127.4), and for CA, it was 18.6 h (min 1.1–max 137.2). The prehospital delay was observed in 545 subjects, of whom 401 (67.8%) presented with CA. The intrahospital delay was observed in 365 patients (39%), with similar percentages in subjects with AA vs. CA (39.8% vs. 38%). The univariable logistic regression model for the likelihood of CA is presented in Table 3. Finally, the multivariate logistic regression model indicated that only young children and prehospital delay were associated with CA (Figure 2).

**Table 3:** Time of delay among patients with acute and complicated appendicitis.

Time of delay	Total *n* = 943	Acute *n* = 352	Complicated *n* = 591	Unadjusted OR (95% CI)	*p*-value
Prehospital delay					
<48 h	398	208 (59)	190 (32)	1.0 (Ref)	0.000
≥48 h	545	144 (41)	401 (68)	3.04 (2.31–4.00)	
Intrahospital delay					
<24 h	578	212 (60)	366 (62)	1.0 (Ref)	0.604
≥24 h	365	140 (40)	225 (38)	0.93 (0.71–1.21)	

Values reported as *n* (%). Odds ratios (ORs) and 95% confidence intervals (CIs) were calculated using univariable logistic regression with complicated appendicitis as the dependent variable.

## 4 Discussion

This study was conducted in a high-volume pediatric hospital that provides surgical care for children without social security in Mexico. It identified a high prevalence of CA that has not changed significantly, even during the COVID-19 pandemic. The proportion was higher than that reported in similar studies worldwide and in a series from Mexico City with comparable characteristics (24). The discrepancy in this outcome could be attributed to the population's social characteristics associated with delays in the diagnosis of appendicitis (25). In 1982, Luckmann R et al. proposed that AA and CA are distinct entities (26); since then, the proportion of CA as a hospital quality indicator for surgical care has been controversial (27). The geographic variability in the incidence rates of AA and CA (3, 28), favorable outcomes to medical treatment (29, 30), and differing gene expression profiles in children CA (31) support the hypothesis that using the proportion of CA as an indicator of institutional quality may not be appropriate.

A key finding in this study was the time elapsed from the onset of symptoms to hospital admission, which is the main risk factor associated with CA. This aligns with the first systematic review by Li et al. (9), which demonstrated that the incidence of CA is related to the total time from symptom onset to either admission or surgery. An updated systematic review by Calpin et al. (1). reported similar results, emphasizing (1) that the risk of complications decreases if patients undergo surgery within the first 24 h after hospital admission. This contrasts with our findings, as hospital delays did not increase the risk of CA This may be attributed to the fact that many patients already had CA due to delays in admission to the emergency department.

According to Bergmark RW (32), two understudied aspects when evaluating access to quality surgical care are when the patient recognizes the need to access the healthcare system and the process of contacting the surgeon who will address their condition. If the main identified risk was prehospital delay, there is sufficient evidence of the association between appendiceal perforation and factors attributable to the patient or those occurring before hospital admission.

Consistent with previous reports, the only risk factor for CA attributable to the patient was being under five years old, which remained significant in the adjusted risk analysis. Some authors associate age with the risk of delayed diagnosis, but the analysis of this population supports the hypothesis of independent risks. In clinical practice, we observe the need for a higher degree of suspicion (25), the difficulty in communicating symptoms, and the variability in the clinical presentation of appendicitis at this age (33). The findings of Dhillon BK regarding a dysregulated immune response in children with CA (31) could support our results.

A relevant limitation of this analysis was the exclusion of antibiotic use before the diagnosis of appendicitis from the risk model. Although we attempted to include this variable, the retrospective design introduced several biases in its measurement, so we decided to exclude it. Antibiotic stewardship is imperative in pediatric perforated appendicitis, although its impact has not yet been well studied (34). In cities such as ours, where we have documented excessive or inappropriate use of antibiotics (35), this factor could modify the evolution of children with appendicitis before they reach specialized care centers.

In children from high-income countries, the association between appendiceal perforation and social vulnerability, as well as barriers to accessing quality surgical care, is strong and consistent (36, 37). In Mexico, we lack comprehensive information on this association, primarily due to fragmented health coverage in both public and private systems and the absence of population data that could help us draw meaningful conclusions. Currently, it is recognized that 96.1% of the Mexican population has access to a hospital with the capacity to perform surgical procedures, based solely on their geographical location (38). However, the reality differs depending on the type of access to healthcare systems and socioeconomic factors associated with social vulnerability. In children from uninsured households, we observed a high frequency of CA, which was related to prehospital delay; consequently, when families contact the surgeon, the children often develop CA.

## Data Availability

The original contributions presented in the study are included in the article/Supplementary Material, further inquiries can be directed to the corresponding author.
